# Management of adult patients with type 1 diabetes mellitus in Africa: A post-hoc cohort analysis of 12 African countries participating in the International Diabetes Management Practices Study (Wave 7)

**DOI:** 10.1097/MD.0000000000020553

**Published:** 2020-06-19

**Authors:** Jean Claude Mbanya, Poobalan Naidoo, Babatope Ayodeji Kolawole, Ellina Tsymbal, Alicia McMaster, Sumanth Karamchand, Hilton Kaplan, Virendra Rambiritch, Mark Cotton, Rachid Malek, Lawrence Allen Distiller, Rory Forseth Leisegang

**Affiliations:** a Medicine and Endocrinology, Faculty of Medicine and Biological Science, University of Yaoundé; b Biotechnology Center and Doctoral School of Life, Health and Environmental Sciences, University of Yaoundé, Yaoundé, Cameroon; c Medical Affairs, Sanofi, Johannesburg, South Africa; d Department of Health Informatics, School of Health Professions, Rutgers, State University of New Jersey, NJ; e Department of Medicine, College of Health Sciences, Obafemi Awolowo University, Ile-Ife, Nigeria; f Department of Medicine, Faculty of Medicine and Health Sciences, Stellenbosch University, Cape Town; g Center of Diabetes and Endocrinology, Johannesburg; h University of KwaZulu-Natal, Biomedical Research Ethics Committee and Discipline of Pharmaceutical Sciences, KwaZulu-Natal; i Family Clinical Research Unit (FAMCRU), Tygerberg Hospital, Department of Pediatrics and Child Health, Stellenbosch University, Stellenbosch, South Africa; j Department of Internal Medicine, CHU Sétif, Algeria; k Clinical Pharmacology & Toxicology, University Hospital / Inselspital Bern, Bern, Switzerland; l Pharmacometrics, Department of Pharmaceutical Biosciences, Uppsala University, Uppsala, Sweden.

**Keywords:** adults, Africa, diabetic keto-acidosis, glycemic control, hypoglycaemia, type 1 diabetes mellitus

## Abstract

There is a paucity of information on real world management of African adult patients with type 1 diabetes mellitus (T1DM). We aimed to describe a cohort of African adults with T1DM.

The International Diabetes Management Practices Study is an observational survey conducted from 2005 to 2017. Data were collected in seven individual waves from countries in Asia, Africa, East Europe, and Latin America. Wave 7 was conducted from 2016 to 2017 and the African cohort included 12 countries. Questionnaires were administered to clinicians and patients. Analyses were mainly descriptive. Logistic regressions were performed to identify predictive factors for glycaemic control.

A total of 788 patients were enrolled in the study. HbA1c values were available for 712 patients; only 16.6% had HbA1c values <7%. A total of 196 (24.9%) reported being hospitalized in the preceding year, with the most common reasons being diabetic ketoacidosis (58.1%, 93/160) and hypoglycaemia (31.1%; 52/167). Over half of the patients (55.4%) stated that the cost of test strips limited regular glycemic monitoring; a minority of patients (15%, 120/788) received structured diabetes education. Predictors of HbA1c <7% included patients receiving diabetes education (odds ratio [OR] [95% confidence interval, CI] = 2.707 [1.157–6.335] *P* = .022), following a healthy diet and exercise plan (OR [95% CI] = 2.253 [1.206–4.209], *P* < .001) and self-managing (monitoring glucose levels and adjusting insulin accordingly) (OR [95% CI] 2.508 [1.500–4.191] *P* < .001).

African adults with T1DM have suboptimal glycemic control with almost one-quarter reporting hospitalization within the preceding year. Most patients felt comfortable with self-adjustment of insulin dose but said that the cost of test strips was the main factor that limited regular monitoring. Reducing direct costs of testing strips and insulin, and improving education will address major challenges within these settings.

## Abstract 2

Supplemental Digital Content is available in the text

## 1 Introduction

Diabetes mellitus is a heterogeneous disorder of carbohydrate, protein, and fat metabolism, characterized by hyperglycemia secondary to defective insulin secretion, insulin action, or both.^[1]^ The majority of patients with diabetes can be classified as either type 1 (T1DM) (5%–10%) or type 2 (90%–95%) diabetes mellitus (T2DM).^[1]^ Although T2DM is more common, the incidence of T1DM is increasing by 2% to 5% per year globally.^[2–5]^ In 2013, an estimated 39,000 patients had T1DM (6.4 per 100,000 children <14 years’ old per year) in the African region.^[6]^ However, estimating the true incidence and prevalence of T1DM in Africa is challenging because epidemiologic studies are limited in number and outdated.^[6–8]^

Notwithstanding advances in medical management of diabetes, patients in the African region continue to encounter challenges in achieving glycemic control but data, including real world evidence studies, are limited.^[6,8]^ Improved glycemic control is associated with a reduction of microvascular and macrovascular complications.^[6,8,9]^ Studies on the of attainment of glycaemic targets in Africa are however scarce.

Given the paucity of information on real world management and challenges experienced by African adult patients with T1DM, we aimed to describe a cohort of adults with T1DM from 12 African countries. We hypothesized that management of African patients with T1DM is suboptimal.

## 2 Methods

### 2.1 Data

The International Diabetes Management Practices Study (IDMPS) is an international, multicenter, observational survey conducted in adult patients diagnosed with T1DM and T2DM.^[10–15]^ The objective of the primary IDMPS study was to evaluate the management of adult patients with T1DM and T2DMin real world settings, and data were collected via questionnaires administered to enrolled clinicians and patients. These data included measures of glycemic control, frequency of HbA1c (glycated hemoglobin) testing, screening for complications of diabetes mellitus, and evaluation of therapy.

From 2005 to 2017, data were collected in 7 individual waves, each of which included a cross-sectional survey. Each wave enrolled participants from countries in Asia, Africa, East Europe, and Latin America.^[14]^ Wave 7 was conducted from 2016 to 2017 and 24 countries participated (Algeria, Bangladesh, Cameroon, Democratic Republic of Congo, India, Iran, Iraq, Ivory Coast, Egypt, Jordan, Kenya, Kuwait, Lebanon, Madagascar, Morocco, Nigeria, Pakistan, Russia, Saudi Arabia, Senegal, South Africa, United Arab Emirates, Tunisia, and Ukraine).^[14]^

This article describes adults with T1DM from 12 African countries that participated in the IDMPS wave 7 study. The number and profile of the physicians who participated in the primary study were determined on a country by country basis. The number of physicians depended on the sample size of patients’ per country. Each physician was requested to enroll the first 10 adults with T2DM and first 5 adults with T1DM visiting during the 2-week recruitment period. To ensure that the participating physicians were representative of the physicians who manage diabetic patients and are experienced in insulin therapy (initiation and titration), a stratified sample was randomly drawn. In Africa, it was planned to select 231 physicians and to recruit 3302 patients with T1DM or T2DM (788 and 2514 with T1DM and T2DM, respectively).

### 2.2 Statistical methods

Qualitative data were summarized using number of non-missing data, number of missing data, counts, and percentages (2-sided confidence interval [CI] 95% of proportion if pertinent); quantitative data were summarized using qualitative descriptive statistics (number of nonmissing data, number of missing data, mean, standard deviations, minimum, and maximum). The statistical analyses were conducted with SAS Software version 9.2 (SAS Institute, Cary, NC).

Logistic regression was used to identify covariates (Supplemental Digital Content Table S1) associated with better glycemic control (HbA1c <7% vs HbA1c ≥7%); missing data were not included in these analyses as the numbers were low and assumed to be missing at random (MAR). Initially, univariate analysis was performed to test for potential predictors listed in Supplemental Digital Content Table S1 (modalities assessed vs reference modality) in association with the dependent variable (HbA1c <7% vs HbA1c ≥7%). Variables significant at a *P* value ≤.10 were included in the full regression model. For the quantitative variables retained at the threshold of 10%, the assumption of log-linearity was assessed. If the assumption of log-linearity was met, then the variable was added in the multivariate model as a quantitative variable; if the assumption of log-linearity was however not met, then the variable was added in the multivariate model as a qualitative variable.

### 2.3 Ethics

The IDMPS study was conducted according to the principles established in the 18^th^ World Medical Assembly and all subsequent amendments, and in accordance with the guidelines for Good Clinical and Epidemiology Practice. Ethics approvals and written informed consent were obtained in each country before initiation and enrolment in the study, respectively.

## 3 Results

### 3.1 Countries

**Figure 1:**
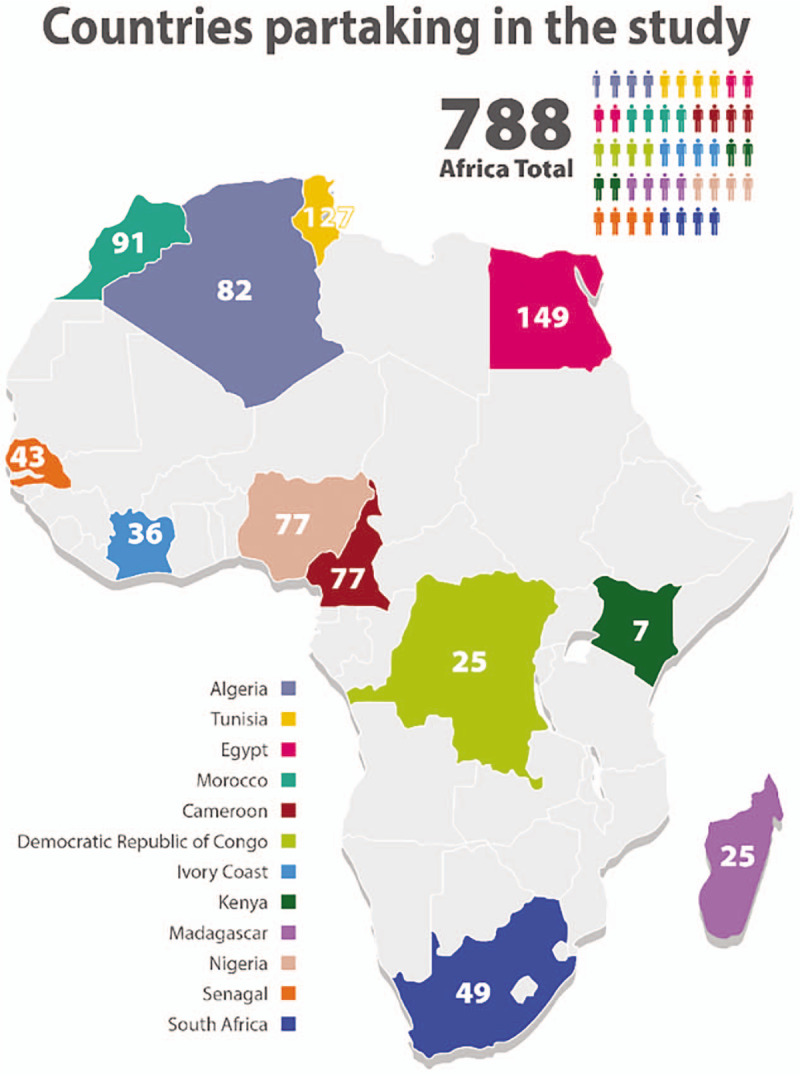
Countries participating in the study.

A total of 814 patients with T1DM were initially included; however, 26 were excluded from the analysis (22 were below the age of 18 years and 4 were not on insulin). The final data set for analysis comprised 788 patients. The countries that participated in the study, and the number of patients enrolled for each country are contained in Figure 1 and including participants form Algeria, Tunisia, Egypt, Morocco, Cameroon, Democratic Republic of Congo (DRC), Ivory Coast, Kenya, Madagascar, Nigeria, Senegal and South Africa; Egypt recruited the majority of patients (149), and Kenya the least (7).

### 3.2 Physician data

In the Africa region, a total of 231 physicians included at least 1 patient in the study: 138 specialized in the management of diabetes (endocrinologists or diabetologists) and 91 were not primarily specialists in diabetes management (general practitioners, primary care practitioners and internists/cardiologists) (information about specialty was not available for 2 physicians). The mean (standard deviation or SD) age of all physicians was 50.6 (9.7) years with 61.9% males. Specialists had been practicing for 22.2 (10.1) years on average and nonspecialists for 23.5 (9.2) years. Majority of the physicians (97.8%) reported following clinical practice guidelines, mainly American Diabetes Association (ADA) or European Association for the Study of Diabetes guidelines.

### 3.3 Social and demographic data

Detailed description of the cohort's social and demographic characteristics is shown in Table 1. Most patients (75.4%) were younger than 40 years with only 16 patients (2%) being older than 65 years; the youngest patient was 18 and the eldest was 83 years of age. Both sexes were equally represented, and patients were mostly white or Black, resided in urban or suburban environment and were literate. The data relating to insurance type were not uniform due to varying systems and terminology used in each of the countries, but 27% of patients reported having to provide an “out of pocket” co-payment for their medication. With regards to employment, 60.8% were in either full or part time employment. Of these 25% took a median of 6 days of sick leave related to diabetes in the past 3 months.

**Table 1:**
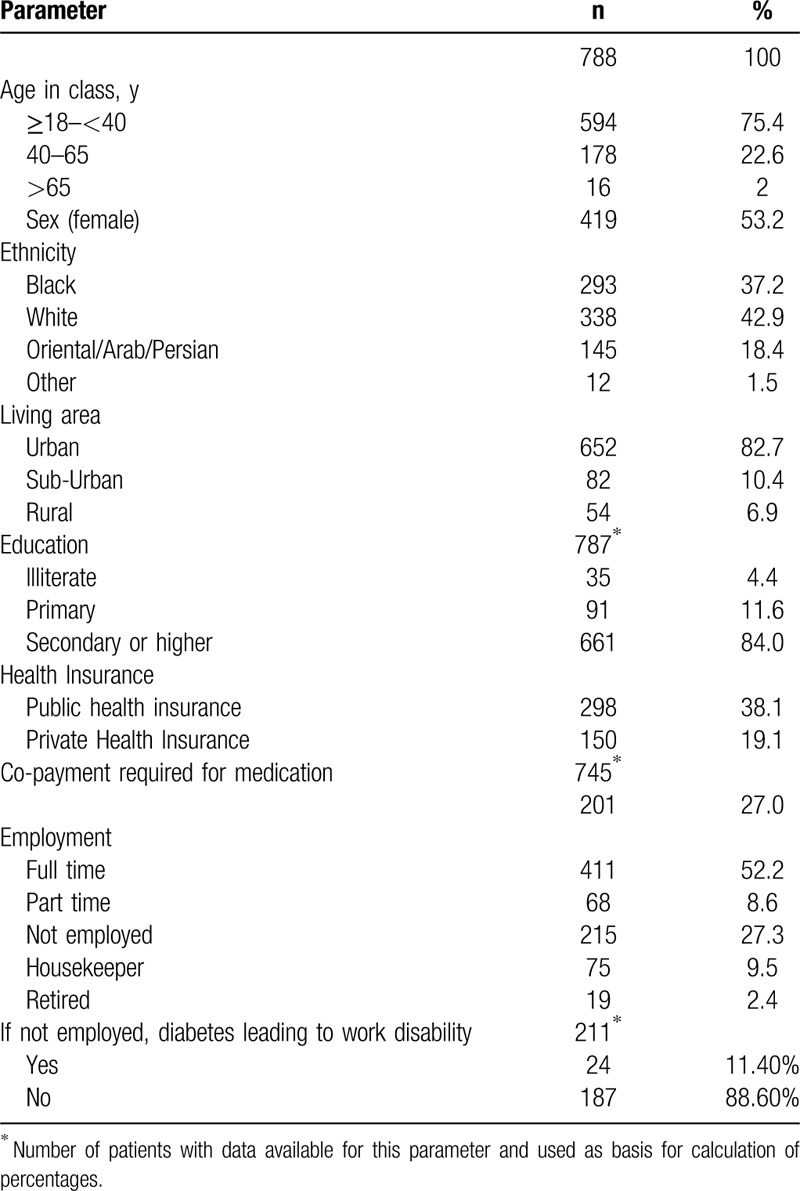
Demographic and social data of the cohort.

### 3.4 Clinical data

Majority (70.1%) of patients had been diagnosed with T1DM for >5 years. Over half of all patients (56.4%) had a body mass index (BMI) <25 kg/m^2^, but 12.2% were obese (BMI >30 kg/m^2^). Median blood pressure was within acceptable limits (systolic blood pressure/diastolic blood pressure = 120/72 mmHg), with 16.5% (130 individuals) previously diagnosed with hypertension.

Lipid profile results were available for less than half of all patients (49.6% for total cholesterol and only 36.5% for low-density lipoprotein (LDL) or LDL-cholesterol). On history, 43 patients reported having familial hypercholesterolemia and another 139 has had some type of dyslipidemia. Approximately 82% of patients have never smoked and 83.9% had either normal or slightly decreased renal function (mean estimated glomerular filtration rate or estimated glomerular filtration rate (SD) of 95.29 (35.89) mL/min/1.73m^2^).

### 3.5 Glycemic monitoring, target achievement, barriers to care and education

**Figure 2:**
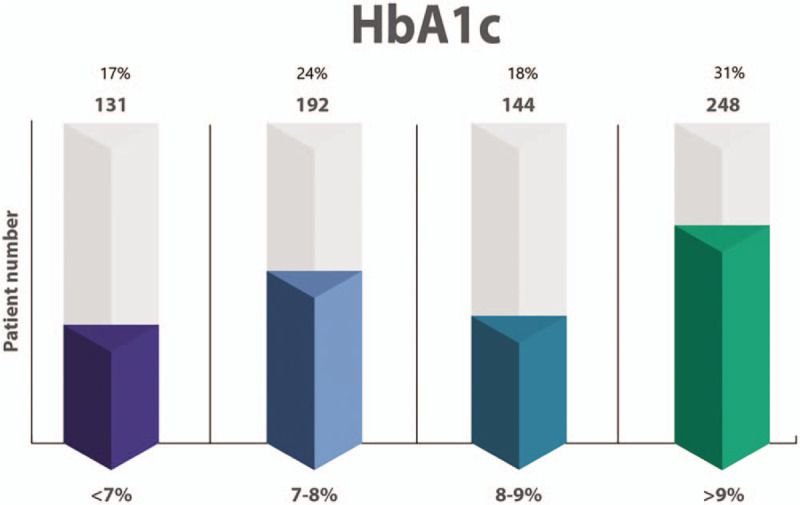
HbA1c of the cohort stratified into 4 categories.

HbA_1c_ values were available for 712 patients and only 16.6% had HbA1c values <7% and 27.8% (215) were recorded as having achieved glycemic target as set by their treating physician (Fig. 2). Only 120 patients (15%) received structured diabetes education. Most patients self-measured fasting blood glucose (FBG) and/or post prandial blood glucose (PPBG), 659 (83.6%) and 414 (52.5%) respectively. Mean (SD) of the last measured FBG was 9.11 (4.46) mmol/L and mean (SD) last PPBG was 11.32 (4.79). Glucometers were available to 79.2% of patients (620), of these 48.7% did self-monitoring of blood glucose (SMBG) daily and 10% “seldom” or “practically never” did them. Over half of the patients (55.4%) reported that the high cost of test strips was the main factor that limited regular monitoring.

**Figure 3:**
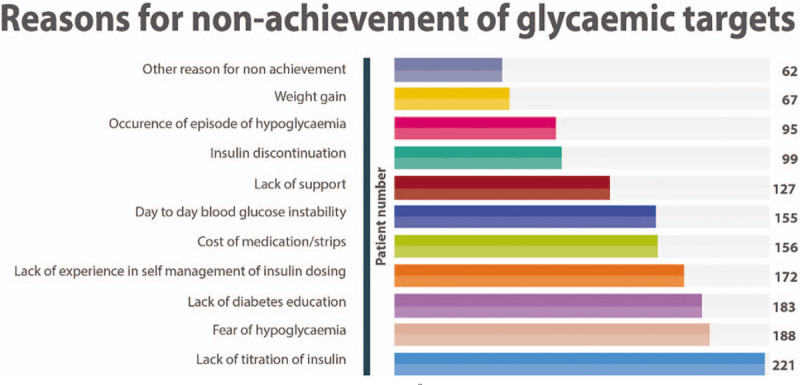
Reasons for nonachievement of glycemic targets. ^∗^More than 1 reason could be selected by the patient.

Patients who did not achieve their targets for HbA1c or self-monitored blood glucose were asked to complete a questionnaire describing the reasons; 554 patients obliged and completed the questionnaire. Key reasons cited by patients for nonachievement of glycemic targets included lack of insulin titration, fear of hypoglycaemia, and lack of diabetes education (Fig. 3).

### 3.6 Insulin type and treatment monitoring

All patients were on insulin therapy and 66 (8.4%) received concomitant oral antidiabetic drugs. In the latter category, 51 were on metformin, 4 on a sulphonylurea, and 3 on metformin plus a sulphonylurea. The remaining 8 patients documented receiving dipeptidyl peptidase-4 inhibitors (DPP4i) (6), alpha-glucosidase inhibitor (1), and glucagon-like peptide-1 receptor agonist (1). Of all the patients on basal and/or prandial insulin, 79.2% and 58.8% used analogue insulins, respectively. The aforementioned is in contrast to patients using premixed insulin, where only 24.6% used an analogue mixture, and the remaining 75.4% used human insulin mixture. Most patients (61.6%) felt comfortable with self-adjustment of insulin dose. Insulin regimen and median total daily doses are shown in the Table 2. A large proportion of patients, irrespective of insulin regimen, did not achieve glycemic targets (Table 2). Furthermore, only 16 patients (2.3% [95% confidence interval, CI 1.3%–3.7%]) achieved triple targets of HbA_1c_ <7%, blood pressure (130/80 mmHg) and LDL-cholesterol <2.6 mmol/L.

**Table 2:**
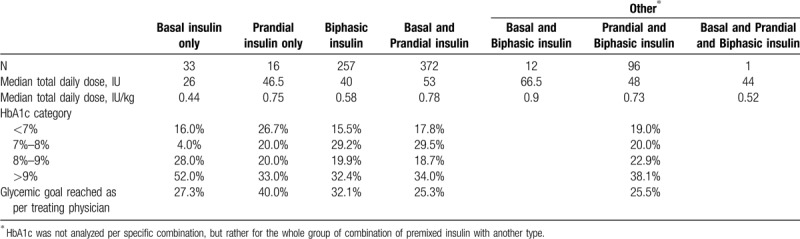
Insulin regimens used and corresponding glycemic control.

### 3.7 Adherence to treatment

**Figure 4:**
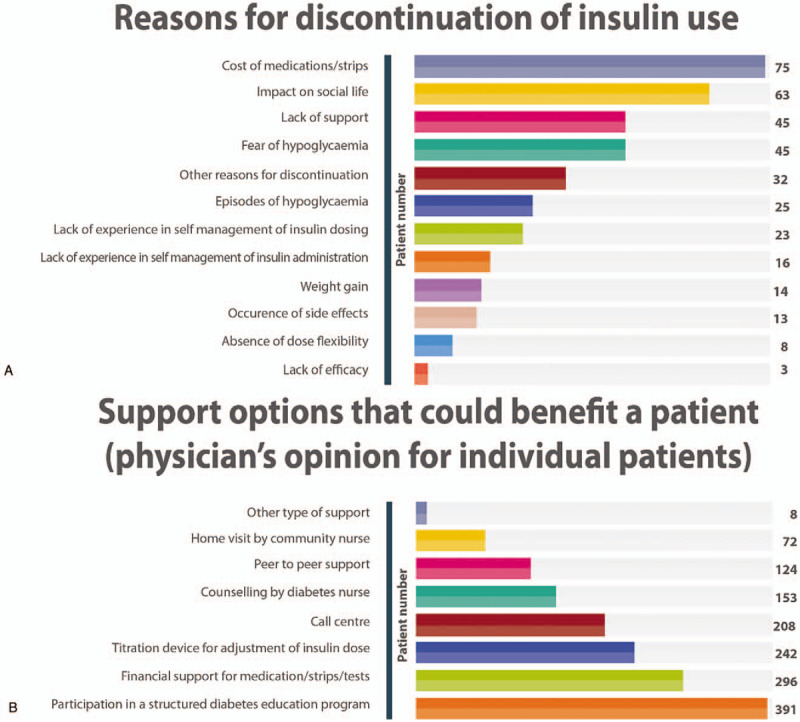
(A) Reasons for discontinuation of insulin use as provided by patients in the questionnaire and (B) support options as per physician's opinion that could have benefitted a patient with regards to adherence to treatment and in achievement of glycemic goal. In both figures, >1 option could have been selected by the patient and the physician.

Although a majority of the patients (83.8%) reported having received some type of diabetes education, only 53.2% confirmed that they adhere to healthy lifestyle and diet. Furthermore, 174 patients reported discontinuing insulin at some time in the past, with duration of discontinuation ranging from few days to >3 years (mean [SD] 2.82 [4.49] months, median 1 month). Common reasons for insulin discontinuation included the cost of medication and/or test strips (43.1%), impact of treatment on social life (36.2%), lack of support (25.9%) and fear of hypoglycaemia (25.9%) (Fig. 4A). According to the treating physicians, most patients (76%) may have benefitted from some type of additional support (Fig. 4B).

### 3.8 Acute complications and hospitalizations

More than half of all the patients (54.3%) reported having experienced a hypoglycemic episode in the previous 3 months and 111 patients (17.9%) had an episode of severe hypoglycemia in the preceding year. Most of these severe episodes were attributed to incorrect insulin usage, most commonly inappropriate dose (53.2%), lack of dose adjustment after exercise (36%), and overestimation of the meal size (25.2%). A total of 196 hospitalizations were reported in the preceding year, with the most common reason being diabetic ketoacidosis (58.1%, 93/160), followed by hypoglycemia (31.1%; 52/167) and admissions for education/initiation/control of diabetes (13.3%; 22/165). The mean (SD) days spent in hospital on account of diabetic ketoacidosis and for education/initiation/control was 8.32 (5.94) and 5.14 (3.41), respectively.

### 3.9 Regression analyses

Univariate logistic regression identified the following as being associated with improved glycemic control (HbA1c <7%): age ≤40 years, BMI ≤25 kg/m^2^, absence of microvascular complication, following a healthy diet and exercise plan, self-monitoring of glucose, self-adjusting insulin, diabetes education, lower total daily insulin dose, less diabetes related hospitalization for past 12 months, and care by specialist. Results are presented in Supplemental Digital Content Table S2.

Multivariate logistic regression identified the following as being associated with improved glycemic control (HbA1c <7%): receiving diabetes education (odds ratio or OR [95% CI] = 2.707 [1.157–6.335] *P* = .022), following a healthy diet and exercise plan (OR [95% CI] 2.253 [1.206–4.209], *P* < .001) and self-managing (monitoring glucose levels and adjusting insulin accordingly) (OR [95% CI] 2.508 [1.500–4.191] *P* < .001). Multivariate logistic regression identified the following as being associated with poor glycemic control (HbA1c ≥7%): hospitalizations due to diabetes during the last 12 months (OR [95% CI] = 2.253 [1.206;4.209], p = 0.011) and duration of diabetes more than 10 years (OR [95% CI] = 1.871 [1.210–2.894], *P* = .005). Results are contained in Supplemental Digital Content Table S3.

## 4 Discussion

Our study findings support our hypothesis that adults with T1DM in Africa are suboptimally managed, with only 17% attaining an HbA_1c_ at or below an acceptable target of 7% and almost 25% reporting being hospitalized in the preceding 12 months.^[16]^ Furthermore, despite the young age of the cohort, one-third had an HbA_1c_ >9%, which predisposes them to development or progression of microvascular and macrovascular complications including diabetic retinopathy, amputations, and myocardial infarction.^[8,16]^ Poor glycemic control predisposes even young patients with T1DM to premature death; a 20-year study in Soweto, South Africa, showed that the mortality in T1DM patients is unacceptably high (20-year mortality of 43%); causes of death included renal failure, hypoglycemia, ketoacidosis).^[17]^

The management of the complications of poor glycaemia are costly and serve as a further strain to limited health care resources in Africa.^[18]^ A study in United Kingdom demonstrated that 80% of costs of % managing patients with diabetes arise from potentially avoidable long-term complications.^[19]^ Our study findings suggest this expenditure could be better spent in preventing long-term complications by improving diabetes education, improving accessibility of therapy and blood glucose test strips, and promoting better self-monitoring of blood glucose control.

The suboptimal attainment of glycemic targets observed in the African T1DM cohort is similar to the results of the primary IDMPS wave 7 study in which only 22% achieved a HbA_1c_ <7%.^[14]^ Physician-reported reasons for nonachievement of glycemic targets include lack of insulin titration, fear of hypoglycaemia, cost of medicine and strips, and lack of diabetes education; these reasons are also similar to the primary IDMPS wave 7 study results,^[14]^ suggesting that developing countries share comparable challenges. Interestingly, our cohort's poor attainment of glycemic targets are similar to the findings of 2 recent studies: a US registry study of 22,697 patients with T1DM in which only 21% of adults with T1DM had an HbA1c <7%^[20]^ and a multinational study (17 countries) of 3858 adults with T1DM in which only 24.3% attained an HbA1c <7%.^[21]^

Although a minority of patients stated their disease as the cause of their unemployment during the study, one-quarter of the employed individuals had taken multiple sick days in the preceding 3 months because of their diabetes. As 60% of this group were in either full or part time employment, development of diabetic complications may have negative socioeconomic repercussions for these patients and their communities. Only half of the patients are managed in accordance with the ADA guidelines which recommend intensive insulin therapy either in the form of multiple daily injections of prandial insulin and basal insulin (basal-bolus regimen) or continuous subcutaneous insulin infusion (CSII).^[16]^ In our cohort, only 7 patients were using the CSII and 372 used the basal bolus regimen. One-third of patients were on biphasic insulin and another 108 patients (13.8%) on a combination of the biphasic insulin and either prandial, basal, or both. The International Diabetes Federation is currently drafting guidelines for the management of patients with T1DM in Africa; given the resource challenges in Africa, their recommendations are eagerly awaited.

Nonadherence to treatment and inadequate monitoring of glycemia was another area of concern; 174 patients (22%) had discontinued insulin at some stage since diagnosed diabetic and 10% admitted to self-monitoring blood glucose only occasionally or seldom. Patients identified cost of the medication and/or testing strip as the major reasons for poor adherence and more is needed to reduce the direct costs. Fear of hypoglycemia and lack of social support and interference with social life were the next most common reasons for insulin discontinuation. This suggests the need for improved education of patients as well as their communities about diabetes and improved access to therapy. It has been shown that patients with T1DM respond to education programs which enable them to self-manage their disease, with improved control of their disease and quality of life.^[16]^ Interestingly, the treating physicians also indicated that, for the majority of patients in the cohort, participation in a structured diabetes education program could help patients reach their glycemic targets. As inadequate insulin titration and fear of hypoglycaemia were stated as the most common causes negatively impacting glycemic control, principles of insulin self-titration, and prevention and management of hypoglycaemia, could be areas of focus for patient education programs.^[22]^

Given that 86% of study subjects have secondary or higher education, it is unlikely likely that literacy was the cause of poor glycemic control, but rather lack of diabetes educational support. The addition of a robust long-term patient support program that includes education on diabetes, lifestyle management, and self-monitoring and insulin titration are likely to get more patients to glycaemic goal given that study subjects were more likely to attain HbA1c of <7% if they received diabetes education, adhered to a healthy diet and exercise plan, and self-managed (monitoring glucose levels and adjusting insulin accordingly). The challenge with long-term patient support programs is cost, but this may be mitigated by the reduced costs associated with good glycemic control and reduced acute and chronic complications.

This is an observational study with populations from diverse and heterogeneous African countries. The different countries from across Africa have different health care systems, and insulin availability and cost vary across the continent. Aggregating data of these patients is an oversimplification without adequate consideration of nuances in individual countries in Africa. There was a disproportionate enrollment of patients from some countries and most of the patients were from urban and sub-urban settings, thus limiting the generalizability of the study findings. The classification of patients as having T1DM was based on the investigators assessment and there is a possibility that patients that were on both oral antidiabetic agents and insulin (8.4%) may have latent autoimmune diabetes in adults or insulin-requiring T2DM. The study sites were private facilities and do not reflect the management of patients in public facilities. The study would have benefitted from assessment of patients’ economic status as this may affect management of diabetes. Furthermore, this study included only 12 of the >50 African countries. Nonetheless, this study provides some indication of the management of African adults with T1DM and adds the growing research in Africa on this topic.^[15]^

## 5 Conclusion

Management of patients with T1DM in Africa is suboptimal. Reasons for suboptimal management include inadequate titration of insulin, fear of hypoglycaemia, inadequate insulin regimens, lack of a structured education programs, and costs. Most patients felt comfortable to do self-adjustment of insulin dose but said that the cost of test strips was the main factor that limited regular monitoring. Clinicians surveyed suggest that care of adults with T1DM may be improved by implementation of structured diabetes mellitus education programs and financial support for antidiabetic therapies and self-monitoring of blood glucose.

## Author contributions

We thank the clinicians and patients partaking in the study. This is a post-hoc analysis and therefore access to patient data, anonymization, and ethics falls with the processes described within the publications.^[10–15]^ In this analysis, Sanofi sponsored the costs related to data extraction, statistical analysis, and article processing fee; no funding was provided for the writing of this manuscript and no medical writing agency was used. All authors interpreted the results, revised the manuscript, and approved the final version of the manuscript. PN is the guarantor of this work and, as such, had full access to all the data in the study and takes responsibility for the integrity of the data and the accuracy of the data analysis. For further queries related to the integrity of the analysis, access to the questionnaires, please contact AM at alicia.mcmaster@sanofi.com.

## Supplementary Material

Supplemental Digital Content
