# Supplementary material for: Management of adult patients with type 1 diabetes mellitus in Africa: A post-hoc cohort analysis of 12 African countries participating in the International Diabetes Management Practices Study (Wave 7)
Source: Medicine (Baltimore). 2020 Jun 19;99(25):e20553. doi: 10.1097/MD.0000000000020553 (PMC7310899; doi:10.1097/MD.0000000000020553)
Supplement: Supplemental Digital Content [file medi-99-e20553-s001.pdf]

Table S1: Potential predictors of HbA1c

Patient's characteristics
o Demography and socio-economic profile:
▪ Age (years and/or class)
▪ Ethnicity (Caucasian / South Asian / Black / Oriental, Arab, Persian / East Asian (Chinese, Japanese, Korean) / South East Asian / Native Latin versus Other)
▪ Health insurance
o Physical examination:
▪ BMI at inclusion (kg/m² and/or class)
o Diabetes medical history
▪ Time since diagnosis (years and/or class)
o Diabetes-related complications
▪ At least one microvascular complication
▪ At least one macrovascular complication
o Diet and life style modification
o Patient diagnosed with dyslipidemia
o SMBG and self-management
▪ Patient self-monitor blood glucose with a glucose meter (if the patient does not have a glucose meter, then this variable will be considered as No)
▪ Self-management
o Diabetes education
▪ Format of the program (Structured courses / Random education (not structured) / Individual / In group)
▪ Frequency of visits to endocrinologist-diabetologist in the past 3 months
o Current treatment:
▪ Current OGLDs therapy (1 OGLD / 2 OGLDs / More than 2 OGLDs versus None)
▪ Current insulin treatment (Basal alone / Prandial alone / Premix alone / Basal + Prandial versus Other)
▪ Total daily dose of insulin (IU/Kg and/or class)
▪ Device (Reusable pen / Disposable pen versus Vials or Pump)
o Hypoglycemia in the past 12 months
▪ Severe episodes of hypoglycaemia (Yes versus No) and number of severe hypoglycaemia
▪ Emergency room visits (or hospitalizations) due to hypoglycemia (number and/or class: Yes versus No)
o Hospitalization due to diabetes in the past 12 months (number and/or class: Yes versus No)
Physician's characteristics:
o Specialty of the physician (Specialists versus Non-specialists)
o Time since the physician began practicing medicine (years and/or class)
o Type of medical structure (Public / Private / Both)

Table S2 (1 of 6): Univariate analyses for identification of predictive factors of HbA1c (<7%/≥7%)

	HbA1c < 7% N=131	HbA1c >= 7% N=584	Total N=715	Test
**Age (years)**				Wilcoxon P = 0.459
N	131	584	715	
Missing	0	0	0	
Mean (SD)	32.89 (12.09)	34.10 (12.53)	33.87 (12.45)	
Median	30.00	30.50	30.00	
Q1;Q3	24 ; 38	24 ; 41	24 ; 40	
Min;Max	18 ; 83	18 ; 75	18 ; 83	
**Age in class (years)**				
N	131	584	715	
<=40	104 (79.4%)	437 (74.8%)	541 (75.7%)	Fisher Exact
]40;65]	22 (16.8%)	139 (23.8%)	161 (22.5%)	P = 0.046
>65	5 (3.8%)	8 (1.4%)	13 (1.8%)	
**Age in class (years)**				
N	131	584	715	
<=65	126 (96.2%)	576 (98.6%)	702 (98.2%)	Fisher Exact
>65	5 (3.8%)	8 (1.4%)	13 (1.8%)	P = 0.071
**Age in class (years)**				
N	131	584	715	
<=40	104 (79.4%)	437 (74.8%)	541 (75.7%)	Chi-2
>40	27 (20.6%)	147 (25.2%)	174 (24.3%)	P = 0.272
**Ethnicity**				
N	131	584	715	
Caucasian	57 (43.5%)	266 (45.5%)	323 (45.2%)	Fisher Exact
South Asian	0	8 (1.4%)	8 (1.1%)	P = 0.738
Black	48 (36.6%)	192 (32.9%)	240 (33.6%)	
Oriental, Arab, Persian	26 (19.8%)	116 (19.9%)	142 (19.9%)	
East Asian	0	0	0	
South East Asian	0	0	0	
Native Latin	0	0	0	
Other	0	2 (0.3%)	2 (0.3%)	
**Ethnicity (4 classes)**				
N	131	584	715	
Caucasian	57 (43.5%)	266 (45.5%)	323 (45.2%)	Chi-2
Black	48 (36.6%)	192 (32.9%)	240 (33.6%)	P = 0.425
Oriental, Arab, Persian	26 (19.8%)	116 (19.9%)	142 (19.9%)	
Other	0	10 (1.7%)	10 (1.4%)	
**Ethnicity (3 classes)**				
N	131	584	715	
Caucasian	57 (43.5%)	266 (45.5%)	323 (45.2%)	Chi-2
Black	48 (36.6%)	192 (32.9%)	240 (33.6%)	P = 0.705
Other	26 (19.8%)	126 (21.6%)	152 (21.3%)	
**Ethnicity (2 classes)**				
N	131	584	715	
Oriental, Arab, Persian	26 (19.8%)	116 (19.9%)	142 (19.9%)	Chi-2
Other	105 (80.2%)	468 (80.1%)	573 (80.1%)	P = 0.997

Data regarding the variable 'HbA1c (<7%/>=7%)' are not available for 73 patients.

Table S2 (part 2 of 6): Univariate analyses for identification of predictive factors of HbA1c (<7%/≥7%)

	HbA1c < 7% N=131	HbA1c >= 7% N=584	Total N=715	Test
**Health Insurance**				
N	131	581	712	
Missing	0	3	3	Chi-2
Yes	75 (57.3%)	331 (57.0%)	406 (57.0%)	P = 0.953
No	56 (42.7%)	250 (43.0%)	306 (43.0%)	
**BMI at inclusion (kg/m²)**				Wilcoxon P = 0.068
N	129	574	703	
Missing	2	10	12	
Mean (SD)	24.25 (3.77)	25.12 (4.70)	24.96 (4.55)	
Median	23.80	24.65	24.50	
Q1;Q3	22 ; 27	22 ; 28	22 ; 28	
Min;Max	16 ; 36	12 ; 50	12 ; 50	
**BMI at inclusion (3 classes)**				
N	129	574	703	
Missing	2	10	12	Chi-2
<= 25	81 (62.8%)	306 (53.3%)	387 (55.0%)	P = 0.018
]25;30]	41 (31.8%)	187 (32.6%)	228 (32.4%)	
> 30	7 (5.4%)	81 (14.1%)	88 (12.5%)	
**BMI at inclusion (2 classes)**				
N	129	574	703	
Missing	2	10	12	Chi-2
<= 25	81 (62.8%)	306 (53.3%)	387 (55.0%)	P = 0.050
> 25	48 (37.2%)	268 (46.7%)	316 (45.0%)	
**Time since diabetes diagnosis (years)**				Wilcoxon P = 0.089
N	131	583	714	
Missing	0	1	1	
Mean (SD)	11.92 (10.61)	13.10 (9.79)	12.89 (9.94)	
Median	8.00	11.00	11.00	
Q1;Q3	4 ; 16	5 ; 19	5 ; 18	
Min;Max	1 ; 61	1 ; 50	1 ; 61	
**Time since diabetes diagnosis in class (years)**				
N	131	583	714	
Missing	0	1	1	Chi-2
<= 5	40 (30.5%)	156 (26.8%)	196 (27.5%)	P = 0.150
]5;10]	35 (26.7%)	124 (21.3%)	159 (22.3%)	
> 10	56 (42.7%)	303 (52.0%)	359 (50.3%)	
**Time since diabetes diagnosis in class (years)**				
N	131	583	714	
Missing	0	1	1	Chi-2
<= 10	75 (57.3%)	280 (48.0%)	355 (49.7%)	P = 0.056
> 10	56 (42.7%)	303 (52.0%)	359 (50.3%)	
**At least one microvascular complication**				
N	121	554	675	
Missing	10	30	40	Chi-2
No	85 (70.2%)	328 (59.2%)	413 (61.2%)	P = 0.024
Yes	36 (29.8%)	226 (40.8%)	262 (38.8%)	

Data regarding the variable 'HbA1c (<7%/>=7%)' are not available for 73 patients.

Table S2 (part 3 of 6): Univariate analyses for identification of predictive factors of HbA1c (<7%/≥7%)

	HbA1c < 7% N=131		HbA1c >= 7% N=584		Total N=715		Test
**At least one macrovascular complication**							
N	121		554		675		
Missing	10		30		40		Chi-2
No	116	(95.9%)	529	(95.5%)	645	(95.6%)	P = 0.854
Yes	5	(4.1%)	25	(4.5%)	30	(4.4%)	
**Patient follow a healthy diet and exercise plan**							
N	131		565		696		
Missing	0		19		19		Chi-2
Yes	106	(80.9%)	271	(48.0%)	377	(54.2%)	P = <0.001
No	25	(19.1%)	294	(52.0%)	319	(45.8%)	
**Patient diagnosed with any other form of dyslipidemia**							
N	122		528		650		
Missing	9		56		65		Chi-2
Yes	20	(16.4%)	115	(21.8%)	135	(20.8%)	P = 0.186
No	102	(83.6%)	413	(78.2%)	515	(79.2%)	
**Patient self-monitor blood glucose with a glucose meter**							
N	130		579		709		
Missing	1		5		6		Chi-2
Yes	115	(88.5%)	435	(75.1%)	550	(77.6%)	P = <0.001
No	15	(11.5%)	144	(24.9%)	159	(22.4%)	
**Patient self-adjust insulin**							
N	131		574		705		
Missing	0		10		10		Chi-2
Yes	102	(77.9%)	345	(60.1%)	447	(63.4%)	P = <0.001
No	29	(22.1%)	229	(39.9%)	258	(36.6%)	
**Patient self-manage (both blood glucose and insulin)**							
N	126		534		660		
Missing	5		50		55		Chi-2
No	30	(23.8%)	234	(43.8%)	264	(40.0%)	P = <0.001
Yes	96	(76.2%)	300	(56.2%)	396	(60.0%)	
**Patient involved in any educational program provided by the physician or his/her clinical staff**							
N	131		584		715		
Yes	114	(87.0%)	499	(85.4%)	613	(85.7%)	Chi-2
No	17	(13.0%)	85	(14.6%)	102	(14.3%)	P = 0.641
**Patient ever received diabetes education**							
N	131		576		707		
Missing	0		8		8		Chi-2
Yes	120	(91.6%)	474	(82.3%)	594	(84.0%)	P = 0.009
No	11	(8.4%)	102	(17.7%)	113	(16.0%)	
**Format of the program**							
N	109		508		617		
Missing	22		76		98		Chi-2
None	11	(10.1%)	102	(20.1%)	113	(18.3%)	P = 0.005
Structured courses	29	(26.6%)	87	(17.1%)	116	(18.8%)	
Random education (not structured)	17	(15.6%)	105	(20.7%)	122	(19.8%)	
Individual	32	(29.4%)	160	(31.5%)	192	(31.1%)	
In group	20	(18.3%)	54	(10.6%)	74	(12.0%)	

Data regarding the variable 'HbA1c (<7%/>=7%)' are not available for 73 patients.

Table S2 (part 4 of 6): Univariate analyses for identification of predictive factors of HbA1c (<7%/≥7%)

	HbA1c < 7% N=131	HbA1c >= 7% N=584	Total N=715	Test
**Follow-up visits by an endocrinologist / diabetologist during the last 3 months in class**				
N	126	530	656	
Missing	5	54	59	Chi-2
None	85 (67.5%)	378 (71.3%)	463 (70.6%)	P = 0.393
>= 1 follow-up visit	41 (32.5%)	152 (28.7%)	193 (29.4%)	
**Frequency of visits to endocrinologist-diabetologist during past 3 months**				Wilcoxon P = 0.283
N	126	530	656	
Missing	5	54	59	
Mean (SD)	0.74 (1.58)	0.54 (1.20)	0.57 (1.28)	
Median	0.00	0.00	0.00	
Q1;Q3	0 ; 1	0 ; 1	0 ; 1	
Min;Max	0 ; 11	0 ; 10	0 ; 11	
**OGLD therapy**				
N	131	584	715	
None	118 (90.1%)	532 (91.1%)	650 (90.9%)	Fisher Exact
1 OGLD	13 (9.9%)	48 (8.2%)	61 (8.5%)	P = 0.665
2 OGLDs	0	4 (0.7%)	4 (0.6%)	
More than 2 OGLDs	0	0	0	
**OGLD therapy (2 classes)**				
N	131	584	715	
None	118 (90.1%)	532 (91.1%)	650 (90.9%)	Chi-2
1 OGLD or more	13 (9.9%)	52 (8.9%)	65 (9.1%)	P = 0.714
**OGLD therapy**				
N	131	584	715	
None	118 (90.1%)	532 (91.1%)	650 (90.9%)	Fisher Exact
1 OGLD	13 (9.9%)	48 (8.2%)	61 (8.5%)	P = 0.665
More than 1 OGLD	0	4 (0.7%)	4 (0.6%)	
**Current insulin treatment**				
N	131	583	714	
Missing	0	1	1	Fisher Exact
No insulin treatment	0	0	0	P = 0.902
Basal alone	4 (3.1%)	21 (3.6%)	25 (3.5%)	
Prandial alone	4 (3.1%)	11 (1.9%)	15 (2.1%)	
Premix alone	40 (30.5%)	176 (30.2%)	216 (30.3%)	
Basal + Prandial	63 (48.1%)	290 (49.7%)	353 (49.4%)	
Other	20 (15.3%)	85 (14.6%)	105 (14.7%)	
**Current insulin treatment**				
N	131	583	714	
Missing	0	1	1	Chi-2
Premix alone	40 (30.5%)	176 (30.2%)	216 (30.3%)	P = 0.926
Basal + Prandial	63 (48.1%)	290 (49.7%)	353 (49.4%)	
Other	28 (21.4%)	117 (20.1%)	145 (20.3%)	

Data regarding the variable 'HbA1c (<7%/>=7%)' are not available for 73 patients.

Table S2 (part 5 of 6): Univariate analyses for identification of predictive factors of HbA1c (<7%/≥7%)

	HbA1c < 7% N=131	HbA1c >= 7% N=584	Total N=715	Test
**Total daily dose (IU/kg)**				Wilcoxon
N	130	577	707	P = <0.001
Missing	1	7	8	
Mean (SD)	0.66 (0.27)	0.77 (0.34)	0.75 (0.33)	
Median	0.63	0.73	0.71	
Q1;Q3	0.49 ; 0.78	0.52 ; 0.96	0.51 ; 0.92	
Min;Max	0.15 ; 1.79	0.02 ; 3.11	0.02 ; 3.11	
**Total daily dose in class (IU/kg)**				
N	130	577	707	
Missing	1	7	8	Chi-2
<0.5 IU/kg	34 (26.2%)	127 (22.0%)	161 (22.8%)	P = 0.309
>=0.5 IU/kg	96 (73.8%)	450 (78.0%)	546 (77.2%)	
**Device used for insulin treatment**				
N	125	568	693	
Missing	6	16	22	Chi-2
Vials or Pump	48 (38.4%)	208 (36.6%)	256 (36.9%)	P = 0.709
Reusable or Disposable Pen	77 (61.6%)	360 (63.4%)	437 (63.1%)	
**Severe episodes of hypoglycaemia (requiring assistance) during the past 12 months**				
N	129	568	697	
Missing	2	16	18	Chi-2
Yes	26 (20.2%)	101 (17.8%)	127 (18.2%)	P = 0.528
No	103 (79.8%)	467 (82.2%)	570 (81.8%)	
**Number of severe hypoglycaemia (requiring assistance) during the past 12 months**				Wilcoxon P = 0.744
N	123	552	675	
Missing	8	32	40	
Mean (SD)	0.85 (3.58)	0.31 (0.92)	0.41 (1.75)	
Median	0.00	0.00	0.00	
Q1;Q3	0 ; 0	0 ; 0	0 ; 0	
Min;Max	0 ; 30	0 ; 10	0 ; 30	
**Emergency room visits (or hospitalizations) due to hypoglycaemia during the past 12 months**				
N	128	549	677	
Missing	3	35	38	Chi-2
No	114 (89.1%)	497 (90.5%)	611 (90.3%)	P = 0.615
Yes	14 (10.9%)	52 (9.5%)	66 (9.7%)	
**Number of emergency room visits (or hospitalizations) due to hypoglycaemia during the past 12 months**				Wilcoxon P = 0.593
N	128	549	677	
Missing	3	35	38	
Mean (SD)	0.24 (0.93)	0.15 (0.51)	0.16 (0.61)	
Median	0.00	0.00	0.00	
Q1;Q3	0 ; 0	0 ; 0	0 ; 0	
Min;Max	0 ; 6	0 ; 4	0 ; 6	

Data regarding the variable 'HbA1c (<7%/>=7%)' are not available for 73 patients.

Table S2 (part 6 of 6): Univariate analyses for identification of predictive factors of HbA1c (<7%/≥7%)

	HbA1c < 7% N=131	HbA1c >= 7% N=584	Total N=715	Test
**Hospitalizations due to diabetes during the past 12 months**				
N	129	571	700	
Missing	2	13	15	Chi-2
Yes	15 (11.6%)	133 (23.3%)	148 (21.1%)	P = 0.003
No	114 (88.4%)	438 (76.7%)	552 (78.9%)	
**Number of hospitalizations due to diabetes during the past 12 months**				Wilcoxon P = 0.004
N	129	571	700	
Missing	2	13	15	
Mean (SD)	0.21 (0.78)	0.36 (0.84)	0.34 (0.83)	
Median	0.00	0.00	0.00	
Q1;Q3	0 ; 0	0 ; 0	0 ; 0	
Min;Max	0 ; 6	0 ; 7	0 ; 7	
**Healthy Diet / exercise AND Hospitalizations**				
N	129	553	682	
Missing	2	31	33	Chi-2
No healthy diet/exercise AND Hospitalizations	6 (4.7%)	56 (10.1%)	62 (9.1%)	P = <0.001
No healthy diet/exercise AND No Hospitalizations	18 (14.0%)	232 (42.0%)	250 (36.7%)	
Healthy diet/exercise AND Hospitalizations	9 (7.0%)	72 (13.0%)	81 (11.9%)	
Healthy diet/exercise AND No Hospitalizations	96 (74.4%)	193 (34.9%)	289 (42.4%)	
**Physician specialty**				
N	129	578	707	
Missing	2	6	8	Chi-2
Specialists	99 (76.7%)	381 (65.9%)	480 (67.9%)	P = 0.017
Non-Specialists	30 (23.3%)	197 (34.1%)	227 (32.1%)	
**Time since the physician began practicing medicine (years)**				Wilcoxon P = 0.961
N	127	576	703	
Missing	4	8	12	
Mean (SD)	22.71 (10.03)	22.56 (9.89)	22.59 (9.91)	
Median	22.00	23.00	23.00	
Q1;Q3	14 ; 31	15 ; 30	15 ; 30	
Min;Max	5 ; 43	1 ; 61	1 ; 61	
**Time since the physician began practicing medicine in class (years)**				
N	127	576	703	
Missing	4	8	12	Chi-2
<= 20 years	57 (44.9%)	254 (44.1%)	311 (44.2%)	P = 0.872
>20 years	70 (55.1%)	322 (55.9%)	392 (55.8%)	
**Type of medical structure**				
N	131	583	714	
Missing	0	1	1	Chi-2
Public Hospital	49 (37.4%)	220 (37.7%)	269 (37.7%)	P = 0.190
Private Clinic	23 (17.6%)	118 (20.2%)	141 (19.7%)	
Private / Office	30 (22.9%)	90 (15.4%)	120 (16.8%)	
Other combinations	29 (22.1%)	155 (26.6%)	184 (25.8%)	

Data regarding the variable 'HbA1c (<7%/>=7%)' are not available for 73 patients.

Table S3: Multivariate analyses for identification of predictive factors of HbA1c (<7%/≥7%) (mixed procedure)

Predictive Factor	Modalities	P-value	Odd Ratio [95% Confidence Interval]
Hospitalizations due to diabetes during the past 12 months	Yes vs No	0.011	2.253 [1.206; 4.209]
Patient ever received diabetes education	No vs Yes	0.022	2.707 [1.157; 6.335]
Patient follow a healthy diet and exercise plan	No vs Yes	<0.001	7.115 [4.299; 11.778]
Patient self-manage (both blood glucose and insulin)	No vs Yes	<0.001	2.508 [1.500; 4.191]
Time since diabetes diagnosis in class (years)	>10 vs ≤10	0.005	1.871 [1.210; 2.894]
